# Computational analysis of the role of mechanosensitive Notch signaling in arterial adaptation to hypertension

**DOI:** 10.1016/j.jmbbm.2022.105325

**Published:** 2022-06-29

**Authors:** Jordy G.M. van Asten, Tommaso Ristori, David R. Nolan, Caitríona Lally, Frank P.T. Baaijens, Cecilia M. Sahlgren, Sandra Loerakker

**Affiliations:** a Department of Biomedical Engineering, Eindhoven University of Technology, Eindhoven, the Netherlands; b Institute for Complex Molecular Systems, Eindhoven University of Technology, Eindhoven, the Netherlands; c School of Engineering and Trinity Centre for Biomedical Engineering, Trinity College Dublin, Dublin, Ireland; d Faculty of Science and Engineering, Biosciences, Åbo Akademi, Turku, Finland

## Abstract

Arteries grow and remodel in response to mechanical stimuli. Hypertension, for example, results in arterial wall thickening. Cell-cell Notch signaling between vascular smooth muscle cells (VSMCs) is known to be involved in this process, but the underlying mechanisms are still unclear. Here, we investigated whether Notch mechanosensitivity to strain may regulate arterial thickening in hypertension. We developed a multiscale computational framework by coupling a finite element model of arterial mechanics, including residual stress, to an agent-based model of mechanosensitive Notch signaling, to predict VSMC phenotypes as an indicator of growth and remodeling. Our simulations revealed that the sensitivity of Notch to strain at mean blood pressure may be a key mediator of arterial thickening in hypertensive arteries. Further simulations showed that loss of residual stress can have synergistic effects with hypertension, and that changes in the expression of Notch receptors, but not Jagged ligands, may be used to control arterial growth and remodeling and to intensify or counteract hypertensive thickening. Overall, we identify Notch mechanosensitivity as a potential mediator of vascular adaptation, and we present a computational framework that can facilitate the testing of new therapeutic and regenerative strategies.

## 1 Introduction

Arteries grow and remodel in response to mechanical stimuli (Humphrey, 2008; Cyron and Humphrey, 2017; Humphrey and Schwartz, 2021). A clinically relevant example is the well-known thickening of arteries in response to hypertension (Wolinsky, 1970; Vaishnav et al., 1990; Matsumoto and Hayashi, 1994; Hayashi and Sugimoto, 2007). It is generally accepted that arteries adapt their morphology and composition to maintain some mechanical quantities at preferred, homeostatic values (Humphrey, 2008; Cyron and Humphrey, 2017; Humphrey and Schwartz, 2021). These quantities are then referred to as mechanical or homeostatic target variables. However, the biological mechanisms underlying this mechano-regulated growth and remodeling (G&R) are not fully understood. In addition, the main mechanical target variables for arterial homeostasis are still debated (Cyron and Humphrey, 2017; Eichinger et al., 2021). Unraveling the biological mechanisms of mechano-regulated arterial adaptation and identifying the main mechanical target variables could provide a better understanding of arterial G&R and cardiovascular diseases, thereby revealing potential treatment strategies or advancing tissue regeneration.

Vascular Smooth Muscle Cells (VSMCs) populating the arterial wall play a crucial role in the regulation of arterial G&R in response to mechanical stimuli (Stanley et al., 2000; O’Callaghan and Williams, 2000; Song et al., 2012; Wanjare et al., 2015). These cells display a remarkable phenotypic plasticity allowing them to switch between contractile and synthetic phenotypes, thereby regulating adaptive G&R processes (Owens et al., 2004; Frismantiene et al., 2018). Contractile VSMCs are generally observed in the homeostatic state of healthy arteries and are responsible for providing muscle tone and vascular functionality (Owens et al., 2004; Frismantiene et al., 2018). Synthetic VSMCs are associated with increased cell proliferation, cell migration, and extra-cellular matrix production and, as such, mainly observed in arteries undergoing active G&R processes (Thyberg et al., 1995; Owens et al., 2004; Rensen et al., 2007; Frismantiene et al., 2018).

**Fig. 1:**
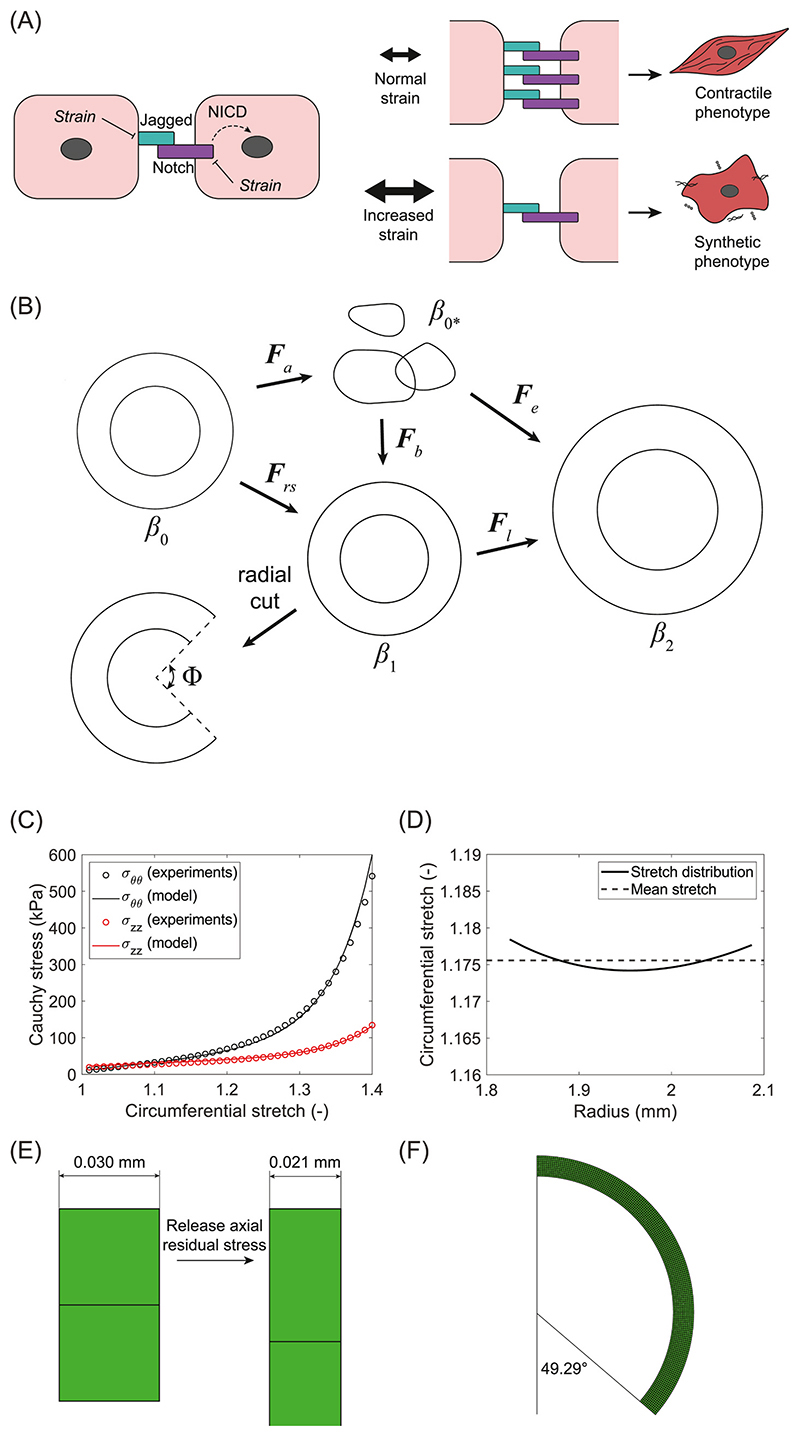
Overview of the methods and processes in the computational framework. (A) Schematic representation of mechanosensitive Notch signaling. Notch receptors of one cell interact with Jagged ligands of an adjacent cell to release NICD which translocates to the nucleus. The expression of Notch3 and Jagged1 is downregulated by mechanical strain. Low strain results in high Notch activity and a contractile phenotype, high strain results in low Notch activity and a synthetic phenotype. (B) Visualization of the kinematics of the simulated coronary artery. (C) Example of the outcome of fitting the model to experimental data of a biaxial tensile test. (D) The circumferential stretch distribution of the arterial wall at normal blood pressure (120/80 mmHg), optimized to be approximately uniform by calibrating the circumferential residual stresses. (E) Releasing the axial residual stresses results in a shortening of the artery in axial direction, consistent with the imposed axial pre-stretch. (F) Releasing the circumferential residual stresses results in the opening of the artery, consistent with the imposed circumferential pre-stretch.

The phenotype of VSMCs is strongly influenced by Notch, a juxtracrine cell-cell signaling pathway that plays a key role in vascular development and homeostasis (Iso et al., 2003; Gridley, 2007; Baeten and Lilly, 2017). The Notch pathway involves interactions between membrane-bound Notch receptors and Jagged or Delta-like ligands (Fig. 1A). Interactions between receptors and ligands of neighboring cells result in the activation of the Notch pathway in the receiving cell. Upon activation, the Notch intracellular domain (NICD) is released and translocates to the nucleus, where it acts as a cofactor for gene transcription, thereby influencing cell behavior (Fig. 1A). In the context of arterial G&R, Notch activation regulates the differentiation of VSMCs from a synthetic to a contractile phenotype (Noseda et al., 2006; Doi et al., 2006; Liu et al., 2009). Contractile and synthetic VSMCs display high and low levels of Notch activation, respectively (Fig. 1A). Notch signaling has also been linked to the adaptive G&R in response to hypertension in both pulmonary and coronary arteries (Li et al., 2009; Qiao et al., 2012; Ragot et al., 2016). However, the mechanisms underlying this involvement of Notch are not fully understood. Interestingly, increasing evidence suggest that the Notch pathway is sensitive to mechanical stimuli (Stassen et al., 2020; Karakaya and Van Asten et al. 2022). In VSMCs, for example, the expression of Notch receptors and ligands is affected by the mechanical strain experienced by these cells (Morrow et al., 2005; Loerakker et al., 2018). We hypothesized that this mechanosensitivity of Notch might be a key phenomenon in arterial adaptation to hypertension, given the important roles of mechanical stimuli and VSMCs in arterial G&R.

Computational models have been extensively adopted to predict the outcomes of various processes and test hypotheses, both in the fields of Notch signaling (reviewed in Binshtok and Sprinzak (2018)) and arterial G&R (reviewed in Ambrosi et al. (2019)). Recently, we have adopted computational models to investigate how Notch mechanosensitivity in VSMCs might regulate arterial G&R (Loerakker et al., 2018; van Engeland et al., 2019; Ristori et al., 2020). However, only simplified mechanical analyses and, consequently, homogeneous strain distributions were adopted in these studies. As the mechanical state of arteries is complex and often heterogeneous, a more detailed analysis of arterial mechanics is necessary to accurately simulate the influence of strain on Notch-regulated phenotypic modulation and G&R.

In this study, we therefore enhanced an existing agent-based (AB) model of mechanosensitive Notch signaling in arteries (Loerakker et al., 2018) with a finite element (FE) analysis of stretches in native coronary arteries subjected to residual stress and hemodynamic loading. The new framework was adopted to investigate the hypothesis that Notch mechanosensitivity could be a mechanism underlying arterial G&R in response to hypertension. For this purpose, three types of hypertension were simulated by increasing either the systolic pressure, diastolic pressure, or both in the FE analysis. As it is still unclear to which specific measure of stretch Notch signaling is responsive, we implemented three hypotheses. We assumed Notch protein expressions to depend on either: i) systolic stretch, i.e. the stretch at systolic pressure with respect to the stress-free configuration, ii) mean stretch, i.e. the stretch at mean blood pressure with respect to the stress-free configuration, or iii) dynamic stretch, i.e. the stretch in the systolic state with respect to the diastolic state. Here, we define mean blood pressure as the sum of the systolic and diastolic pressure divided by two (based on (Braeu et al., 2017, 2019)). We found that the mechanosensitivity of Notch could explain the experimentally observed thickening of arteries in response to three different types of hypertension only when mean stretch was chosen as the target variable. We also studied the effects of variations in residual stress and manipulations in the Notch pathway on VSMC phenotype in hypertension, as targeting Notch signaling has previously been suggested as a potential therapeutic target in hypertension (Morris et al., 2019) and other vascular diseases (Zhou et al., 2015; Zhu et al., 2017; Davis et al., 2018; Ristori et al., 2021). The model predicted that changes in the expression of Notch receptors had a bigger effect on VSMC phenotype than changes in the expression of Jagged ligands. Such changes in Notch expression enabled the modulation of VSMC phenotype and could thereby intensify or counteract the G&R response to hypertension. In the future, Notch signaling models could provide a mechanistic basis for computational G&R frameworks.

## 2 Methods

A computational framework was developed by combining a FE analysis of the mechanical behavior of coronary arteries with an AB model of mechanosensitive Notch signaling. Previous studies have shown such a coupling between FE and AB models to be feasible (Nolan and Lally, 2018; Keshavarzian et al., 2018). Briefly, in the FE model, a native coronary artery was modeled as a residually stressed, fiber-reinforced cylinder exposed to blood pressure. To describe the mechanical behavior of arterial tissue accurately, the mechanical parameters were fitted to experimental data (Van Den Broek et al., 2011). The FE model was coupled to a previously developed AB Notch model, consisting of a system of ordinary differential equations describing the influence of mechanical stimuli on Notch signaling between VSMCs in the arterial wall. In this section, both models and the one-way coupling between them are discussed in more detail.

### 2.1 Finite element analysis of arterial mechanics

#### 2.1.1 Kinematics

The kinematics of the simulated artery are visualized in Fig. 1B. The starting point was the initial, stress-free configuration *β*_0_. Residual stresses in both axial and circumferential directions were subsequently included via deformation gradient tensor ***F****_rs_*, resulting in an unloaded, but residually stressed configuration *β_1_. **F**_rs_* was split into tensor ***F***_*a*_, resulting in an incompatible, stress-free configuration *β*_0_*, and elastic tensor ***F***_*b*_ to ensure compatibility of the material:

(1) $$F_{rs}=F_{b}F_{a}$$

Tensor ***F***_*b*_ was thus responsible for the residual stresses in the material. The residually stressed configuration *β*_1_ was subsequently subjected to loading by blood pressure resulting in the deformation described by ***F***_l_. Finally, we considered the total elastic deformation gradient tensor ***F***_*e*_:

(2) $$F_{e}=F_{l}F_{b}$$

representing the deformation from the stress-free, incompatible configuration *β*_0_* to the loaded configuration *β*_2_. This elastic deformation ***F***_*e*_ was thus responsible for all the stresses in the material. We define the following tensors with respect to a cylindrical coordinate system:

(3) $$F_{rs}=\text{diag}\left[\lambda_{rs}^{r},\lambda_{rs}^{\theta },\lambda_{rs}^{z}\right]$$

(4) $$F_{a}=\text{diag}\left[\lambda_{a}^{r},\lambda_{a}^{\theta },\lambda_{a}^{z}\right]$$

(5) $$F_{b}=\text{diag}\left[\lambda_{b}^{r},\lambda_{b}^{\theta },\lambda_{b}^{z}\right]$$

(6) $$F_{e}=\text{diag}\left[\lambda_{e}^{r},\lambda_{e}^{\theta },\lambda_{e}^{z}\right]$$

where superscripts *r*, *θ*, and *z* represent the radial, circumferential, and axial directions, respectively.

#### 2.1.2 Constitutive model

The material of the coronary artery was modeled as a fiberreinforced, slightly compressible, hyperelastic solid. The strain energy density function was split into an isotropic matrix part (Ψ_*m*_) and an anisotropic fiber part (Ψ_*f*_):

(7) $$\text{Ψ}={\text{Ψ}}_{m}+{\text{Ψ}}_{f}$$

The constitutive behavior of the isotropic matrix was described by a Neo-Hookean model:

(8) $${\text{Ψ}}_{m}=\left(1-\varphi_{f}\right)\left[\frac{\kappa }{2}\ln^{2}\left(J_{e}\right)+\frac{\mu }{2}\left(I_{1,e}-3-2\ln\left(J_{e}\right)\right)\right]$$

with the bulk modulus κ estimated as:

(9) $$\kappa =\frac{2\mu (1+\nu )}{3(1-2\nu )}$$

and

(10) $$I_{1,e}=C_{e}:I$$

Here, *φ_f_* is the total volume fraction of fibers in the material, κ the bulk modulus, *J_e_* the volume ratio of the total elastic deformation (*J_e_* = det (***F**_e_*)), *μ* the shear modulus, *v* the Poisson’s ratio, ***C***_*e*_ the right Cauchy-Green tensor of the total elastic deformation $\left(C_{e}=F_{e}^{T}F_{e}\right)$, *I*_1_,*e* the first invariant of ***C***_*e*_, and ***I*** the second-order unit tensor.

A total of *N* fiber directions was considered, each given as $e_{0}^{i}=\cos\left(\omega^{i}\right)e_{\theta }+\sin\left(\omega^{i}\right)e_{\text{z}}$ in configuration *β_0_* and assumed to lie in the circumferential-axial plane with angle *ω^i^* relative to the circumferential direction, where *i* = 1, 2, …, *N*. The volume fraction of each fiber direction was determined with the following periodic version of the normal probability distribution (Gasser et al., 2006; Driessen et al., 2008):

(11) $$\varphi_{f}^{i}=B\exp\left[\frac{\cos\left(2\left(\omega^{i}-\alpha \right)\right)+1}{d}\right]$$

with *a* the main fiber angle, *d* the dispersity, and *B* a scaling factor to ensure that the sum of the individual fiber volume fractions equals *φ_f_*. The strain energy density function of the fibers in each direction was given by an exponential relationship, following (Oomen et al., 2018):

(12) $${\text{Ψ}}_{f}^{i}=\frac{k_{1}}{2k_{2}}\left(\exp\left[k_{2}\left({\left(\lambda_{e}^{i}\right)}^{2}-1\right)\right]-k_{2}\left({\left(\lambda_{e}^{i}\right)}^{2}-1\right)-1\right)$$

where *k*_1_ and *k*_2_ are material parameters, and $\lambda_{e}^{i}$ is the elastic fiber stretch associated with the total elastic deformation:

(13) $$\lambda_{e}^{i}=\sqrt{{\text{C}}_{e}:\left({\text{e}}_{0^{*}}^{i}⊗{\text{e}}_{0^{*}}^{i}\right)}$$

Here, ${\text{e}}_{0}^{i}$ indicates the direction of the fibers in configuration *β*_0_*: ${\text{e}}_{0^{*}}^{i}=\frac{{\text{F}}_{a}\cdot {\text{e}}_{0}^{i}}{{\text{F}}_{a}\cdot {\text{e}}_{0}^{i}}$ with $e_{0}^{i}$ the initial fiber direction in *β*_0_. The fibers were assumed to bear stress only under tension $\left(\lambda_{e}^{i}>1\right)$. The total strain energy density of the fibers was obtained by taking the sum over the number of fiber directions:

(14) $${\text{Ψ}}_{f}=\overset{i=1}{\underset{N}{\sum^{\text{​}}}}\varphi_{f}^{i}{\text{Ψ}}_{f}^{i}$$

#### 2.1.3 Model parameters

The material parameters were based on experimental data from porcine coronary arteries (Van Den Broek et al., 2011). A constitutive model was derived from these (Van Den Broek et al., 2011) and adopted here to simulate two uniaxial and five biaxial tensile tests and generate stress-strain data. This collection of tensile tests was chosen to obtain a material model that is able to accurately capture the biaxial deformations that arteries are subjected to under normotensive and hypertensive loading. The material parameters of our constitutive model (section 2.1.2) were subsequently fitted to these stress-strain data by solving a non-linear least-squares problem using the Levenberg-Marquardt algorithm in Matlab R2021b (Mathworks, Natick, MA, USA). The results of the fitting procedure are shown in Fig. S1 and a representative example is shown in Fig. 1C. The fitted curves successfully represented the mechanical behavior of porcine coronary arteries, as demonstrated by the average coefficient of determination of 0.98, ranging from 0.94 to 1.00 (see Fig. S1). The coefficient of determination was defined as $R^{2}=1-\sum^{\text{​}}{\left(\sigma_{\exp}-\sigma_{fit}\right)}^{2}/\sum^{\text{​}}{\left(\sigma_{\exp}-{\bar{\sigma }}_{\exp}\right)}^{2}$ with *σ_exp_* and *σ_fit_* the experimental and fitted Cauchy stresses, respectively, and ${\bar{\sigma }}_{exp}$ the mean of the experimental Cauchy stresses. Initial guesses for the material parameters were varied and finally chosen to maximize the coefficient of determination. Modest deviations in the values of these initial guesses (up to 30% above and 10% below these guesses) did not affect the resulting material parameters, indicating that a local minimum was found which was sufficient as the aim was to capture the material behavior and not to identify unique material parameters.

The axial residual stress was assumed to be homogeneous over the arterial wall. Its magnitude was based on the average axial pre-stretch observed in porcine coronary arteries (Van Den Broek et al., 2011): *λ_axial_* = 1.39. To incorporate the residual stresses associated with this axial pre-stretch, parameter $\lambda_{a}^{z}$ was chosen as the inverse of *λ_axial_*, based on the fact that the axial length of the arterial segment remained constant in the simulations via boundary conditions. In other words: $\lambda_{rs}^{z}$ was equal to 1, so in order for $\lambda_{b}^{z}$ to equal the desired value of *λ_axial_*, $\lambda_{a}^{z}$ must be $\lambda_{\text{axial }}^{-1}$ As only the elastic tensor ***F***_*b*_ causes residual stresses in the material, the resulting residual stresses in axial direction are equivalent to those that would be present after a pre-stretch of *λ_axial_*.

A radial cut in an unloaded, excised segment of a native artery typically causes the segment to open at a certain opening angle Φ (Fig. 1B), revealing the presence of residual stress in the circumferential direction. The associated circumferential pre-stretch *λ_circ_* describes the deformation from the opened, stress-free geometry to the closed, residually stressed configuration. It is often believed that this circumferential residual stress acts to homogenize the circumferential stress distribution in an artery under physiological loading (Chuong and Fung, 1986; Fung, 1991). Our aim was therefore to achieve such a homogeneous stress distribution of the loaded artery in the model by implementing residual stress in circumferential direction. The circumferential residual stress was included by choosing a suitable value for parameter $\lambda_{a^{\theta }}^{\theta }$ Similar to the axial case, $\lambda_{a^{\theta }}^{\theta }$ was assumed to be the inverse of the circumferential pre-stretch *λ_circ_*. Two common assumptions in arterial modeling are that the opened, stress-free geometry is a circular segment and that the arterial thickness remains constant (Holzapfel et al., 2000; Humphrey, 2002). With these assumptions, the circumferential pre-stretch *λ_circ_*, as a function of the radius, may be calculated (Holzapfel et al., 2000; Humphrey, 2002):

(15) $$\lambda_{circ}(r)=\frac{kr_{1}}{r_{0}}$$

with

(16) $$k=\frac{2\pi }{2\pi -\text{Φ}}$$

Here, *r_1_* is the radius in the unloaded configuration and *r*_0_ is the corresponding radius in the opened, stress-free configuration. The values for *λ_circ_(r)* thus depend on the opening angle Φ. We subsequently varied the value of the opening angle until the resulting values for *λ_circ_*(*r*) caused an approximately homogeneous circumferential stress distribution over the arterial wall in the loaded configuration. This optimization was performed by minimizing the following objective function:

(17) $$f=\overset{p=1}{\underset{N_{p}}{\sum^{\text{​}}}}\left(\left|{\left(\lambda_{e}^{\theta }\right)}^{p}-{\vec{\lambda }}_{e}^{\theta }\right|\right)$$

Here, ${\left(\lambda_{e}^{\theta }\right)}^{p}$ are the elastic circumferential stretch ratios, associated with ***F***_*e*_, in each integration point *p* obtained from a FE simulation, ${\bar{\lambda }}_{e}^{\theta }$ is the mean stretch ratio, and *N_p_* the number of integration points. The simulations to obtain ${\left(\lambda_{e}^{\theta }\right)}^{p}$ were performed using a coronary artery that was residually stressed, both in axial and circumferential directions, and loaded with a mean physiological pressure of 100 mmHg. This revealed that the optimal stress distribution was reached with an opening angle of 98.9° and corresponding linear profile for *λ_circ_* of 0.9715 on the inside and 1.0247 on the outside of the arterial wall. The resulting elastic stretch distribution ${\left(\lambda_{e}^{\theta }\right)}^{p}$ is shown in Fig. 1D. Given the uniform materialproperties, the stress distribution was also approximately homogeneous, fulfilling our main aim for including the circumferential residual stress.

To verify the implementation of the axial residual stress, an excision of the axially residually stressed artery was simulated. This allowed the residual stress in axial direction to be released and resulted in a shortening of the arterial segment from 0.03 mm to 0.021 mm (Fig. 1E), consistent with the imposed axial pre-stretch of 1.39. Similarly, a radial cut in the circumferentially residually stressed artery was simulated to verify the correct implementation of the circumferential residual stress. This resulted in the artery opening at an angle of 98.58° (twice the value of 49.29° in the half cylinder in Fig. 1F), very close to the calibrated optimal opening angle. This confirms that both residual stresses were correctly included in the arterial model.

#### 2.1.4 Finite element implementation

The geometry of the coronary artery was modeled as a quarter section of a cylinder using symmetry boundary conditions in circumferential direction on the two open surfaces whose normal vector lies in circumferential direction. Additional boundary conditions in axial direction were imposed on the two circular ends of the cylinder section to prevent all displacements in the axial direction. The dimensions of the artery were based on those of porcine coronary arteries and are given in Table 1. The thickness *T_0_* of the arterial wall in the stress-free configuration was obtained from the literature (Van Den Broek et al., 2011). The inner radius in the stress-free configuration, *r*_*i*,0_, was set such that the inner radius in the unloaded configuration approximately matched the value estimated from the pressure-radius plots in (Van Den Broek et al., 2011). A mesh consisting of 957 quadratic brick elements with reduced integration (C3D20R) was created. Radial pressure was applied to the inner surface of the artery. The analysis was performed in Abaqus 6.14 (Dassault Systèmes Simulia Corp., Providence, RI, USA) and the constitutive model including residual stress was implemented via a custom UMAT subroutine.

### 2.2 Notch signaling model

#### 2.2.1 Notch signaling kinetics

A one-dimensional AB model recently developed by Loerakker et al. (2018), based on (Sprinzak et al., 2011; Boareto et al., 2015), was adopted to simulate the kinetics of the Notch signaling pathway between VSMCs. Due to symmetry in the vascular wall, a one-dimensional array of VSMCs in radial direction was considered. As endothelial cells line the lumen of coronary arteries, one endothelial cell was modeled to be present on the luminal side of the first VSMC in the one-dimensional array. This endothelial cell provided a constant source of Jagged ligands (*J_EC_)* available for interacting with the Notch receptors on the first VSMC. Other Notch proteins on the endothelial cell were not considered.

For each VSMC, the protein content of Notch, Jagged, Delta, and NICD was tracked over time with a set of ordinary differential equations. This set of equations considered the production and degradation of the proteins, trans-interactions between receptors and ligands of adjacent cells, and cis-interactions between receptors and ligands of the same cell:

(18) $$\frac{dN_{j}}{dt}=N_{pr}H^{S}\exp\left(A_{N}E_{\theta \theta ,j}\right)-k_{c}N_{j}\left(D_{j}+J_{j}\right)-k_{t}N_{j}\left(\frac{D_{j-1}+D_{j+1}+J_{j-1}+J_{j+1}}{2}\right)-\gamma N_{j}$$

(19) $$\frac{dJ_{j}}{dt}=J_{pr}H^{S}\exp\left(A_{J}E_{\theta \theta ,j}\right)-k_{c}J_{j}N_{j}-k_{t}J_{j}\left(\frac{N_{j-1}+N_{j+1}}{2}\right)-\gamma J_{j}$$

(20) $$\frac{dD_{j}}{dt}=D_{pr}H^{S}-k_{c}D_{j}N_{j}-k_{t}D_{j}\left(\frac{N_{j-1}+N_{j+1}}{2}\right)-\gamma D_{j}$$

(21) $$\frac{dI_{j}}{dt}=k_{t}N_{j}\left(\frac{D_{j-1}+D_{j+1}+J_{j-1}+J_{j+1}}{2}\right)-\gamma_{I}I_{j}$$

Here, *Nj, Jj, Dj,* and *Ij* are the Notch, Jagged, Delta, and NICD content of cell *j* = 1*,* 2, …, *N_c_*, respectively, with *N_c_* the number of cells based on the vessel wall thickness *T*_0_ and assuming a cell size of 0.01 mm. *N_pr_*, *J_pr_*, and *D_pr_* are the base production rates of Notch, Jagged, and Delta. Parameters *k_c_* and *k_t_* indicate the strength of cis- and trans-interactions, respectively. Degradation of Notch, Jagged, and Delta was assumed to occur at the same rate *γ,* and the degradation rate of NICD was given by *γ_I_.* The mechanosensitivity of Notch and Jagged was accounted for by multiplying the base production rates with the exponential terms *exp*(*A_N_E_θθ,j_*) and exp(*A_J_Eθθ,j*). Here, *A_n_* and *A_j_* are the mechanosensitivity parameters determined in Loerakker et al. (2018), and *E_θθ,j_* is the normal Green-Lagrange strain in circumferential direction in cell *j* (see equation (23)).

The effects of transactivation on the production of Notch, Jagged, and Delta were included with the following Hill function:

(22) $$H^{S}(I,\text{Λ},n)=\text{Λ}+\frac{1-\text{Λ}}{1+{\left(\frac{I}{I_{0}}\right)}^{n}}$$

with Λ defining the changes in protein production in response to Notch transactivation (upregulation for Λ *>* 1, downregulation for Λ *<* 1, and no change for Λ = 1). The parameter *I*_0_ defines the transition point of the Hill function from convex to concave and the parameter *n* determines the sensitivity of protein production to the NICD content. Polarized clustering of Jagged, the preferential positioning of Jagged ligands on the abluminal side of the VSMCs, was considered in the model of Loerakker et al. (2018) but not included here. It had only minor effects and was therefore neglected, similar to Ristori et al. (2020).

#### 2.2.2 Parameters, boundary, and initial conditions

The initial values for the protein content of Notch, Jagged, Delta, and NICD for each cell were randomly generated between 0 and 6000 molecules, similar to Loerakker et al. (2018) and Ristori et al. (2020). Each simulation was repeated 25 times and all simulations converged to the same steady state solution after a simulated time of 250 h, independent of the initial protein content. As mentioned in section 2.2.1, the first VSMC (*j* = 1) of the one-dimensional array can interact with an EC which is assumed to possess a constant value of Jagged ligands (*J_EC_*), and no Notch receptors or Delta ligands. Therefore, the boundary conditions for VSMC *j* = 1 are *J*_*j*−1_ = *J_EC_* and *N*_*j*−1_ = *D*_*j*−1_ = 0. The last VSMC (*j* = *N_c_*) is assumed to interact only with the VSMC with index *j* = *N_c_* – 1; thus, the boundary conditions for the VSMC with index *j* = *N_c_* are: *N*_*j*+1_ = *D*_*j*+1_ = *J*_*j*+1_ = ^0^.

The number of VSMCs in the arterial wall was calculated using the thickness of the coronary artery in the stress-free configuration assuming a VSMC size of 0.01 mm. This number was assumed to remain constant in all simulations, so cell proliferation or apoptosis were not considered. All other parameter values were taken from previous studies (Boareto et al., 2015; Loerakker et al., 2018; Ristori et al., 2020) (Table 1). Details on the biological and experimental motivation of these parameters can be found in (Boareto et al., 2015 Supporting Information S1) and (Ristori et al., 2020 Appendix).

#### 2.2.3 Phenotype prediction

The Notch signaling activity, quantified by the number of NICD proteins in the steady state solution of the set of equations 18-21, was used to predict a phenotype for each VSMC. In previous models (Boareto et al., 2015; Loerakker et al., 2018; van Engeland et al., 2019; Ristori et al., 2020), fixed threshold values of NICD content were used to categorize VSMCs as either a sender, sender/receiver, or receiver cell. Subsequently, a sender state was associated with a synthetic phenotype, and a sender/receiver state with a contractile phenotype. Here, we adopted a different approach. We first determined the NICD content of the VSMCs in a healthy artery at normal blood pressure, referred to as the reference state. We assumed that the VSMCs exhibit a homeostatic, contractile phenotype at this NICD level in the reference state. Changes in the NICD level in subsequent simulations were then used to determine a shift in VSMC phenotype. Specifically, a decrease in NICD content was assumed to result in a more synthetic VSMC phenotype, while an increase in NICD content was assumed to lead to a more contractile phenotype. These assumptions were based on the experimental observation that Notch activation results in the differentiation of VSMCs to a more contractile phenotype (Noseda et al., 2006; Doi et al., 2006; Liu et al., 2009). This new method was motivated by the suggestion that there exists a continuous spectrum of VSMC phenotypes, with the synthetic and contractile phenotypes representing the extremes (Chamley-Campbell et al., 1979; Shanahan and Weissberg, 1998).

### 2.3 One-way coupling

The two models were coupled by taking the stretch distribution in the artery obtained in the FE analysis to inform mechanosensitive Notch signaling between VSMCs in the AB model. It was assumed that the VSMCs are mainly loaded in the circumferential direction. Therefore, the circumferential stretch ratios were collected from the FE simulations. The definition of the circumferential stretch ratio that was used, depended on the chosen target variable for Notch. For systolic stretch or mean stretch as target variables, the elastic stretch ratio $\lambda_{e}^{\theta }$ was used that describes the deformation from the incompatible, stress-free configuration *β*_0_* to the loaded configuration *β_2_* at systolic or mean pressure, respectively. When dynamic stretch was chosen as the target variable, $\lambda_{e}^{\theta }$ under systolic loading was divided by $\lambda_{e}^{\theta }$ under diastolic loading. Then, the resulting stretch ratios in the integration points were redistributed over the number of VSMCs in the AB Notch model to obtain the circumferential stretch in each cell, *λ_j_*. Finally, these were converted to the normal Green-Lagrange strain in circumferential direction and used as input for equations (18) and (19):

(23) $$E_{\theta \theta ,j}=\frac{1}{2}\left(\lambda_{j}^{2}-1\right)$$

## 3 Results

### 3.1 Notch mechanosensitivity can explain arterial G&R in hypertension with mean stretch as target variable

First, the new computational framework was adopted to investigate whether the mechanosensitivity of Notch signaling might regulate the thickening of arteries in response to hypertension. We considered three types of hypertension: isolated systolic hypertension (ISH), isolated diastolic hypertension (IDH), and combined hypertension (CH), where both the systolic and diastolic pressure are increased. The blood pressure values chosen for each hypertension type were based on commonly used guidelines (Whelton et al., 2018). For each type of hypertension, we calculated the radial distribution of systolic stretch, mean stretch, and dynamic stretch in the arterial wall for several combinations of systolic and diastolic blood pressure values. The stretch distributions were then used as input for the simulation of Notch signaling and, consequently, the prediction of the VSMC phenotypes in the arterial wall. Previous experimental studies have demonstrated that all three types of hypertension result in a thickening of arteries (Al-Nimer et al., 2010; Chen et al., 2011; Manios et al., 2015; Monzo et al., 2021). A synthetic phenotype of VSMCs, inducing arterial G&R, is therefore expected in all these situations.

**Fig. 2:**
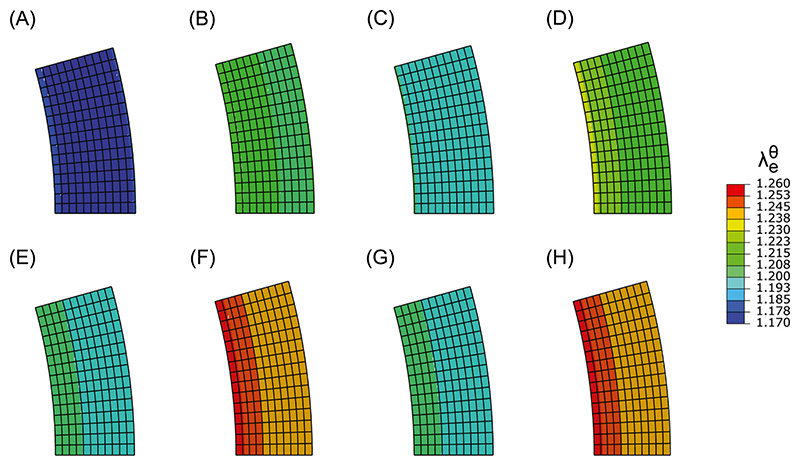
Distributions of the elastic circumferential stretch over the arterial wall for different blood pressure values. (A) mean stretch at normal blood pressure, (B) mean stretch in ISH, (C) mean stretch in IDH, (D) mean stretch in CH, (E) systolic stretch at normal blood pressure, (F) systolic stretch in ISH, (G) systolic stretch in IDH, (H) systolic stretch in CH.

**Fig. 3:**
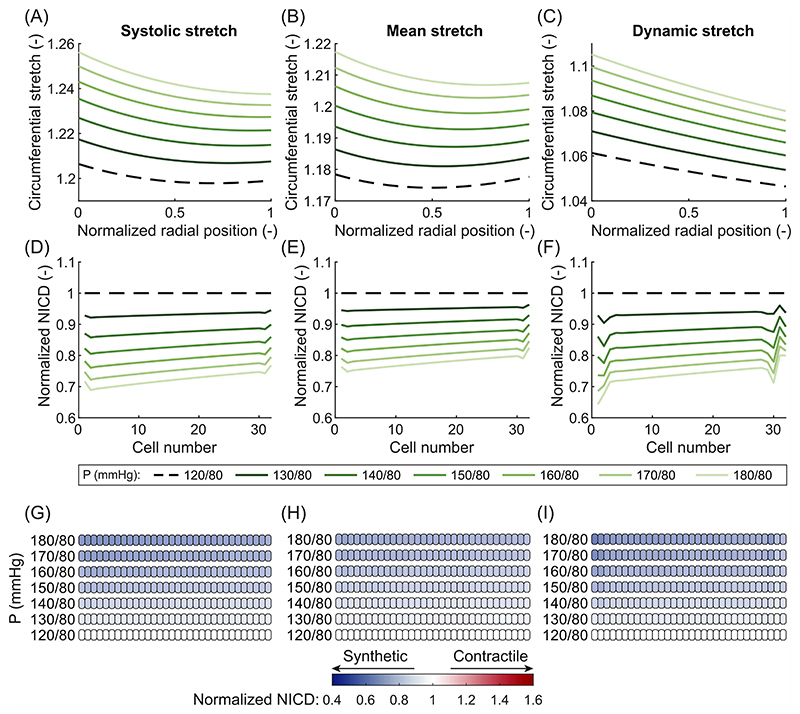
Results of the computational framework for different blood pressure values associated with isolated systolic hypertension. Elastic circumferential stretch distributions over the arterial wall of systolic stretch (A), mean stretch (B), and dynamic stretch (C). Predicted NICD levels of the VSMCs in the arterial wall based on the stretch distributions using systolic stretch (D), mean stretch (E), or dynamic stretch (F) as target variable for mechanosensitive Notch signaling. NICD values are normalized to the situation at normal blood pressure (dashed line). Predicted phenotypes of the VSMCs in the arterial wall based on their NICD content using systolic stretch (G), mean stretch (H), or dynamic stretch (I) as target variable for mechanosensitive Notch signaling. White cells represent homeostatic VSMCs under normal blood pressure, blue cells represent progressively more synthetic VSMCs, and red cells represent progressively more contractile VSMCs. In all figures, the arterial wall runs from the luminal side on the left to the outside on the right.

In case of ISH, VSMCs were predicted to become more synthetic compared to the normotensive state, irrespective of the chosen measure of stretch. In particular, isolated systolic hypertension caused an increase of all three measures of stretch throughout the entire arterial wall (Fig. 2B,F and 3A-C). Following this increase in stretch, the expression of Jagged and Notch decreased, leading to fewer Notch interactions between the VSMCs. As a result, the model predicted a decrease in NICD content, indicating a lower level of Notch activation in all cases (Fig. 3D-F). This, in turn, caused a change in the predicted phenotype of the VSMCs towards the synthetic state (Fig. 3G–I). Therefore, Notch mechanosensitivity can directly explain arterial G&R in response to ISH, irrespective of the chosen measure of stretch.

**Fig. 4:**
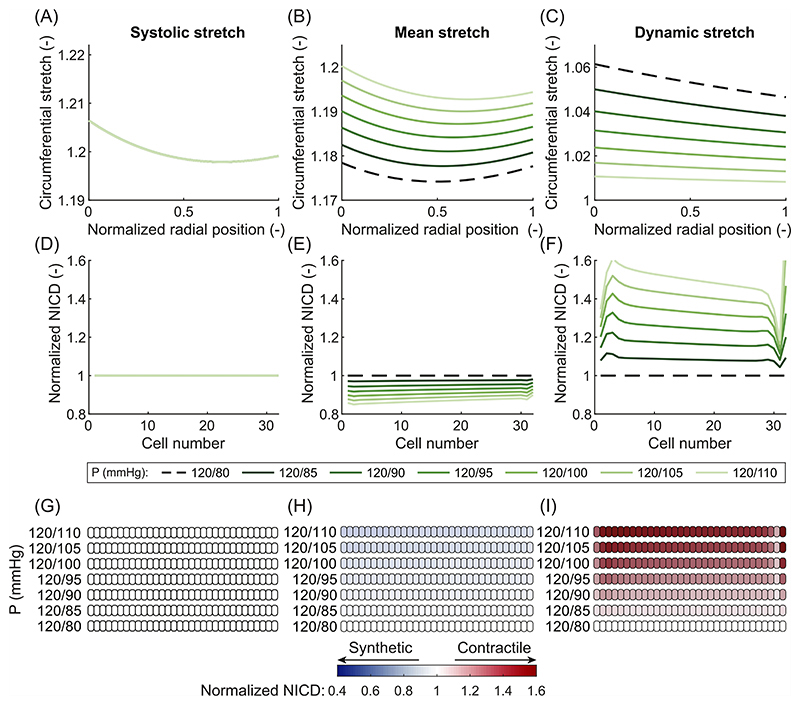
Results of the computational framework for different blood pressure values associated with isolated diastolic hypertension. Elastic circumferential stretch distributions over the arterial wall of systolic stretch (A), mean stretch (B), and dynamic stretch (C). Predicted NICD levels of the VSMCs in the arterial wall based on the stretch distributions using systolic stretch (D), mean stretch (E), or dynamic stretch (F) as target variable for mechanosensitive Notch signaling. NICD values are normalized to the situation at normal blood pressure (dashed line). Predicted phenotypes of the VSMCs in the arterial wall based on their NICD content using systolic stretch (G), mean stretch (H), or dynamic stretch (I) as target variable for mechanosensitive Notch signaling. White cells represent homeostatic VSMCs under normal blood pressure, blue cells represent progressively more synthetic VSMCs, and red cells represent progressively more contractile VSMCs. In all figures, the arterial wall runs from the luminal side on the left to the outside on the right.

In contrast, the effects of IDH were different depending on the chosen stretch hypothesis. In particular, IDH led to no change in the systolic stretch (Figs. 2G and 4A); an increase in the mean stretch (Figs. 2C and 4B); and a sharp decrease in the dynamic stretch (Fig. 4C). This occurred because the diastolic pressure was increased while the systolic pressure remained at normal levels. Therefore, the mean load on the artery increased, while the systolic load stayed the same, explaining the increase in mean stretch and the unchanged systolic stretch. This also caused the difference between diastolic and systolic pressure (i.e. the pulse pressure) to decrease, causing the reduction in dynamic stretch. Selecting systolic stretch as the target variable resulted in no changes in the NICD content (Fig. 4D) or VSMC phenotype (Fig. 4G) upon IDH. In contrast, the increase in mean stretch led to a decrease in NICD content (Fig. 4E) and a change to a more synthetic VSMC phenotype (Fig. 4H), similar to ISH. Finally, the decrease in dynamic stretch led to a higher content of Jagged and Notch and, consequently, a higher level of Notch activity. This resulted in an increase in NICD content (Fig. 4F) and a corresponding shift to a more contractile VSMC phenotype (Fig. 4I). These simulations therefore revealed that, under IDH, an increase in arterial thickness can be predicted only when Notch was assumed to be mechanosensitive to the mean stretch.

**Fig. 5:**
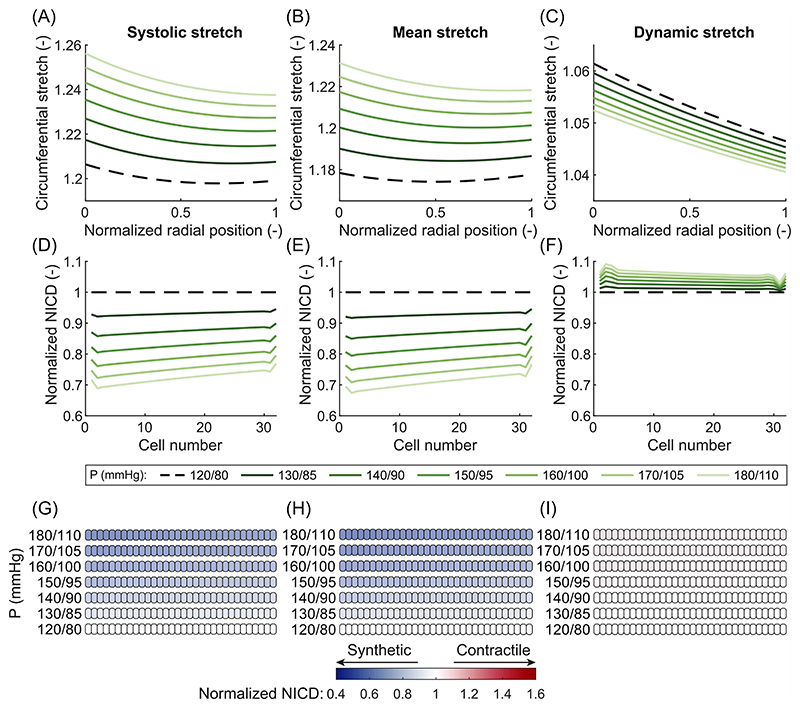
Results of the computational framework for different blood pressure values associated with combined hypertension. Elastic circumferential stretch distributions over the arterial wall of systolic stretch (A), mean stretch (B), and dynamic stretch (C). Predicted NICD levels of the VSMCs in the arterial wall based on the stretch distributions using systolic stretch (D), mean stretch (E), or dynamic stretch (F) as target variable for mechanosensitive Notch signaling. NICD values are normalized to the situation at normal blood pressure (dashed line). Predicted phenotypes of the VSMCs in the arterial wall based on their NICD content using systolic stretch (G), mean stretch (H), or dynamic stretch (I) as target variable for mechanosensitive Notch signaling. White cells represent homeostatic VSMCs under normal blood pressure, blue cells represent progressively more synthetic VSMCs, and red cells represent progressively more contractile VSMCs. In all figures, the arterial wall runs from the luminal side on the left to the outside on the right.

CH resulted in yet another outcome dependent on the chosen stretch hypothesis. Imposing CH on the arteries resulted in an increase in mean (Figs. 2D and 5A) and systolic stretch (Figs. 2H and 5B), but a slight decrease in dynamic stretch (Fig. 5C). This can be explained by the fact that both the diastolic and systolic pressures were increased, causing the artery to extend in both the mean and systolic states. The decrease in dynamic stretch was caused by a combination of two effects. First, while the absolute pulse pressure increased, the increase in the relative pulse pressure was less strong due to the overall higher pressure levels. Second, the artery’s non-linear mechanical behavior resulted in a stiffer response to the pulse pressure at these higher pressure levels and consequently smaller differences in stretch between the systolic and diastolic configurations. These changes in stretch were followed by a decrease in NICD content and a change towards more synthetic VSMCs only when systolic or mean stretch were selected as target variables for Notch (Fig. 5D, E, G, and H). Choosing dynamic stretch as target variable caused an increase in NICD content and slightly more contractile VSMCs (Fig. 5F and I). Taken together, these results show that arterial thickening in response to CH can only be explained by Notch mechanosensitivity when mean or systolic stretch are chosen as target variable for Notch.

Together, the simulations predicted a shift to a more synthetic phenotype following each type of hypertension only when Notch was assumed to depend on the mean stretch. Therefore, the results suggest that Notch mechanosensitivity can provide an explanation for arterial G&R in response to hypertension if mean stretch is selected as the target variable. Since other studies have also found Notch signaling to be involved in arterial G&R in hypertension (Li et al., 2009; Qiao et al., 2012; Ragot et al., 2016), we conclude that mean stretch is the most likely target variable for mechanosensitive Notch signaling. Consequently, for all following simulations, we assumed that Notch signaling was sensitive to the mean stretch.

### 3.2 A decrease in residual stress enables arterial G&R and causes a more heterogeneous phenotype distribution

The level of detail of the FE model featured in the new computational framework enables the investigation of the effects of several mechanical parameters on Notch signaling and VSMC phenotype. Here, for example, we investigated the effects of changes in axial and circumferential residual stress, both with and without hypertension, on Notch signaling and the corresponding VSMC phenotype.

**Fig. 6:**
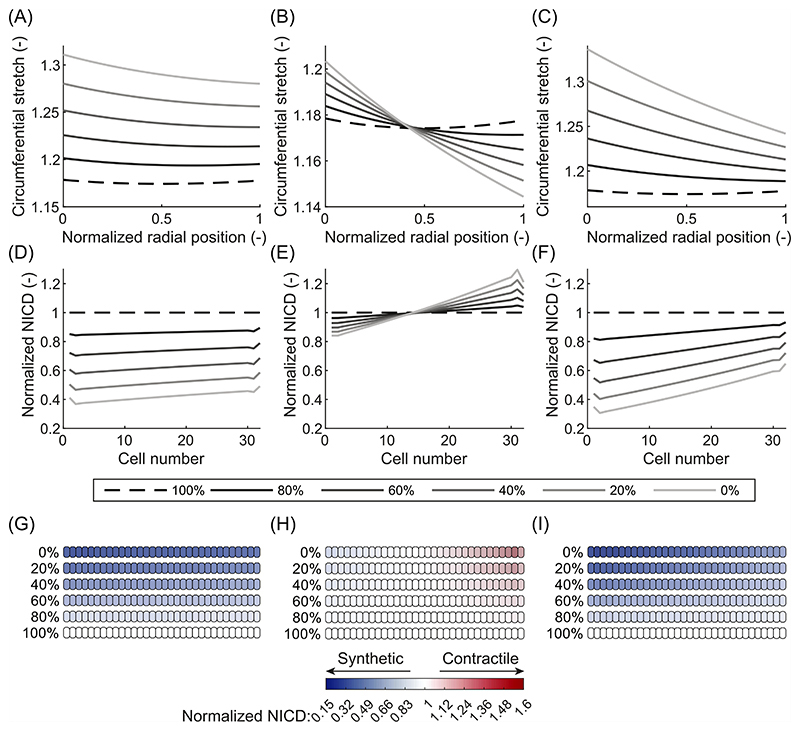
Effects of a loss of residual stress on circumferential stretch, NICD content, and VSMC phenotype. Elastic circumferential stretch distributions over the arterial wall at mean blood pressure for a loss of axial residual stress (A), circumferential residual stress (B), and a combination of both (C). Predicted NICD levels of the VSMCs in the arterial wall for a loss of axial residual stress (D), circumferential residual stress (E), and a combination of both (F). Predicted phenotypes of the VSMCs in the arterial for a loss of axial residual stress (G), circumferential residual stress (H), and a combination of both (I). White cells represent homeostatic VSMCs, blue cells represent progressively more synthetic VSMCs, and red cells represent progressively more contractile VSMCs. The percentages indicate the amount of residual stress present in the material. In all figures, the arterial wall runs from the luminal side on the left to the outside on the right. Percentages indicate the amount of axial and/or circumferential pre-stretch that was lost.

**Fig. 7:**
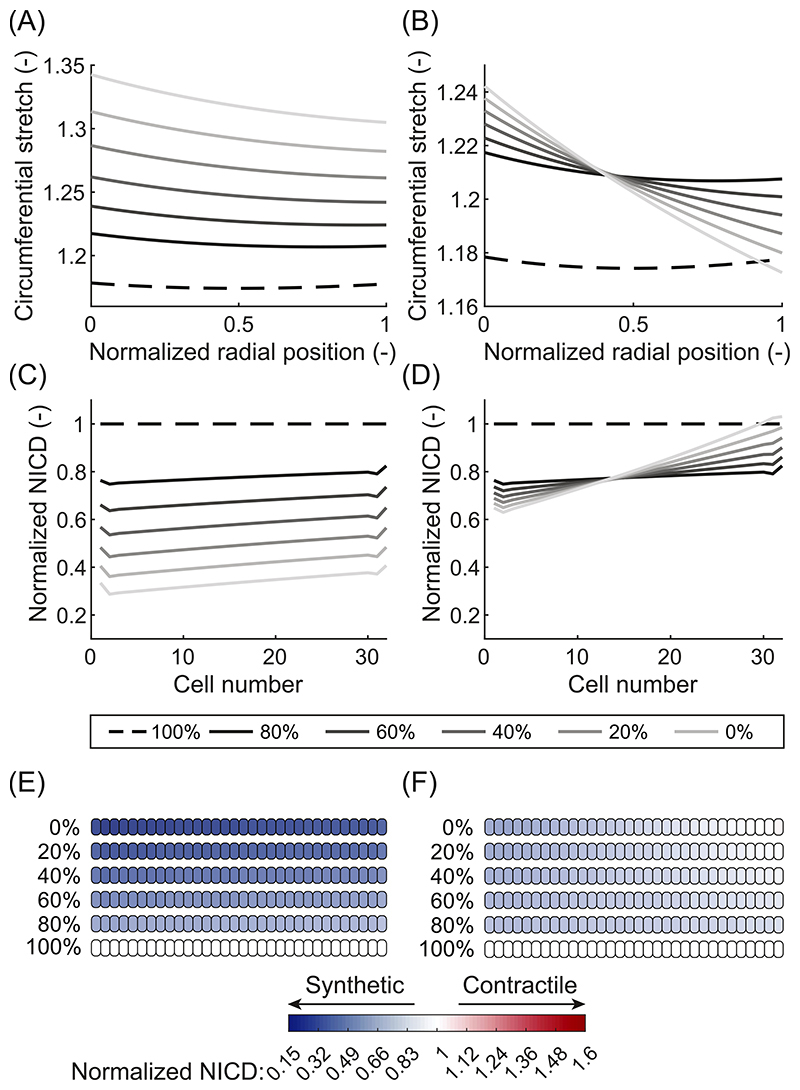
Effects of a loss of residual stress combined with hypertension on circumferential stretch, NICD content, and VSMC phenotype. Elastic circumferential stretch distributions over the arterial wall at mean blood pressure for a loss of axial (A) and circumferential residual stress (B). Predicted NICD levels of the VSMCs in the arterial wall for a loss of axial (C) and circumferential residual stress (D). Predicted phenotypes of the VSMCs in the arterial wall for a loss of axial (E) and circumferential residual stress (F). White cells represent homeostatic VSMCs, blue cells represent progressively more synthetic VSMCs, and red cells represent progressively more contractile VSMCs. The percentages indicate the amount of residual stress present in the material. In all figures, the arterial wall runs from the luminal side on the left to the outside on the right. Percentages indicate the amount of axial or circumferential pre-stretch that was lost.

First, a decrease in the axial residual stress was simulated. This resulted in higher mean stretches (Fig. 6A), followed by a lower NICD content (Fig. 6D), and therefore a shift to more synthetic VSMCs (Fig. 6G), similar to the effects of hypertension. The increase in mean stretch is caused by the material becoming stiffer at higher strains due to its non-linear mechanical behavior. The reduction in axial residual stress then brought the artery into a less stiff region of its mechanical response, thus increasing the mean stretch under load. These results suggest that hypertension and a loss of axial residual stress might have synergistic effects on arterial thickening. To investigate this hypothesis, we performed additional simulations in which a decrease in axial residual stress was accompanied by combined hypertension. This resulted in a stronger shift to synthetic VSMCs (Fig. 7A,C,E), thereby confirming the possible synergy. Overall, these simulations reveal that a loss of axial residual stress results in a more synthetic phenotype of VSMCs, inducing arterial G&R.

Next, a loss of circumferential residual stress was simulated by lowering the opening angle value used to calculate the imposed circumferential pre-stretch *λ_circ_*(*r*) (equation (15)). This loss of circumferential residual stress caused a less uniform distribution of the mean stretch (Fig. 6B), which resulted in a more heterogeneous distribution of NICD content over the arterial wall (Fig. 6E). As a consequence, a broader range of VSMC phenotypes was predicted, with synthetic cells on the inside of the artery and contractile cells on the outside (Fig. 6H). When a loss of circumferential residual stress and hypertension were simulated simultaneously, VSMCs on the inside of the artery were predicted to become most synthetic and therefore have a greater contribution to adaptive G&R (Fig. 7B,D,F). These results demonstrate that a loss of circumferential residual stress can cause regional differences in VSMC phenotype and arterial G&R, with higher G&R activity towards the luminal side of the artery.

Finally, a loss of both axial and circumferential residual stress resulted in a combination of the previously observed effects (Fig. 6C, F, and I). In particular, the loss of axial residual stress caused a generally more synthetic phenotype of the VSMCs, while the loss of circumferential residual stress resulted in regional differences of VSMC phenotype in the arterial wall. Specifically, VSMCs on the inside of the arterial wall were predicted to become more synthetic than those towards the outside. Overall, these simulations show that a simultaneous loss of axial and circumferential residual stress is predicted to induce arterial G&R, especially towards the luminal side of the arterial wall.

Taken together, these simulations demonstrate that a loss of axial residual stress results in a shift of the VSMCs to a more synthetic phenotype, inducing G&R, while a loss of circumferential residual stress creates a more heterogeneous distribution of VSMC phenotypes over the arterial wall. When axial and circumferential residual stress are both decreased, all VSMCs in the artery were predicted to become synthetic, with the most synthetic cells on the luminal side, creating the highest G&R activity there.

### 3.3 Notch manipulations can increase or counteract the effects of hypertension

Finally, we investigated the effects of manipulations to the Notch pathway on VSMC phenotype in hypertensive arteries. These simulations were motivated by previous studies in which Notch manipulations were investigated as potential treatment options in hypertension (Morris et al., 2019) and other cardiovascular diseases (Zhou et al., 2015; Zhu et al., 2017; Davis et al., 2018; Ristori et al., 2021). As an example, following experimental studies of Notch and Jagged knockdown or overexpression (Morrow et al., 2005; Driessen et al., 2018), we considered changes in the cellular expression of Notch receptors and Jagged ligands by altering the basal Notch or Jagged production rates, *N_pr_* and *J_pr_*, in equations (18) and (19). The FE model was adopted to obtain the mean stretch distribution for arteries under the most severe case of combined hypertension (Fig. 5B). This stretch distribution was subsequently used as input for the Notch AB model with altered basal production rates.

**Fig. 8:**
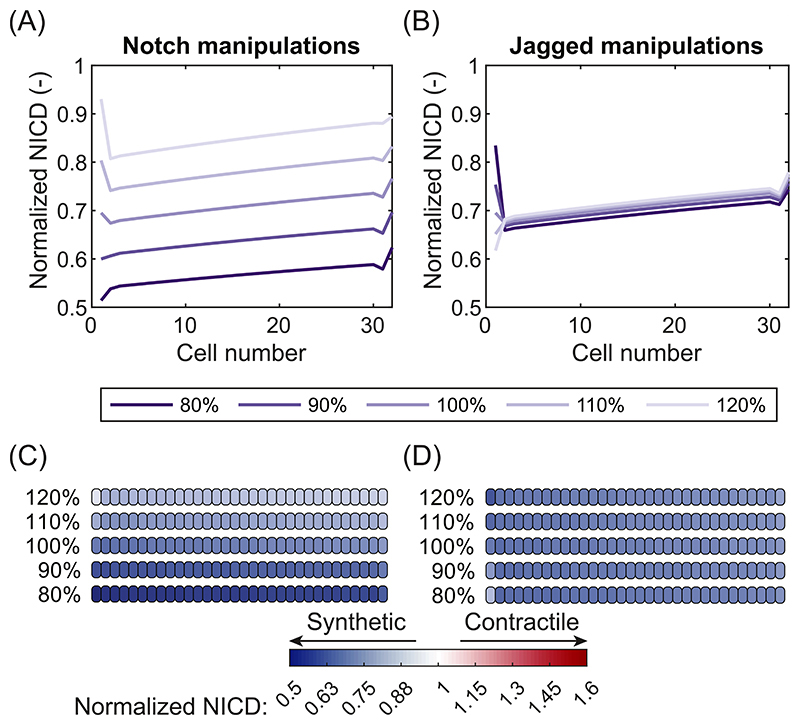
Effects of Notch manipulations on NICD content and VSMC phenotype. Predicted NICD levels of the VSMCs in the arterial wall for changes in Notch expression (A) and Jagged expression (B). Predicted phenotypes of the VSMCs in the arterial wall for changes in Notch expression (C) and Jagged expression (D). White cells represent homeostatic VSMCs, blue cells represent progressively more synthetic VSMCs, and red cells represent progressively more contractile VSMCs. In all figures, the arterial wall runs from the luminal side on the left to the outside on the right. Percentages indicate the relative Notch or Jagged expression levels.

Relatively small changes of Notch expression were predicted to have a considerable influence on NICD content and VSMC phenotype (Fig. 8A, C). In particular, the model predicted that a decrease in Notch receptor expression lowers the NICD content and causes a shift to a more synthetic phenotype, thus intensifying the effects of hypertension on the VSMC phenotype. On the other hand, an increase in Notch expression counteracted the effects of hypertension by increasing NICD content and partly restoring the VSMCs back to their homeostatic phenotype. These results can be explained by the fact that a change in the production rate of Notch altered the number of Notch receptors available for activation by Jagged ligands on neighboring cells. In short, the simulations indicate that changing the expression of Notch receptors may be an effective way of regulating VSMC phenotype which might be used to counteract or intensify the artery’ s G&R response to hypertension.

In contrast, the effects of similar changes in the expression of Jagged ligands were far less substantial (Fig. 8B,D). The NICD content and VSMC phenotype only changed very slightly when an increase or a decrease in Jagged expression was simulated. This outcome occurred because the Notch receptors were already saturated by an excess of Jagged ligands in the reference state. Increasing or decreasing the production of Jagged therefore had very little effect on the number of Notch receptors binding to Jagged ligands. These results suggest that Notch signaling in arteries is more sensitive to changes in Notch expression than to changes in Jagged expression.

Based on these simulations, we can conclude that manipulations to the Notch pathway may be a promising tool to exercise some control over VSMC phenotype and thus arterial G&R. These manipulations may find interesting applications in the treatment of arterial diseases, but also in the field of tissue engineering. In that case, our simulations suggest that it may be more effective to target Notch receptors than Jagged ligands, as the phenotype of VSMCs was predicted to be more sensitive to changes in Notch expression.

## 4 Discussion

Unraveling the mechanisms of mechano-mediated arterial G&R is fundamental to improve our understanding of vascular diseases and identify new treatment strategies. Here, we adopted a multiscale computational approach to investigate the mechanisms of arterial thickening in response to hypertension. The Notch signaling pathway is known to be involved in this process (Li et al., 2009; Qiao et al., 2012; Ragot et al., 2016), but the underlying mechanisms remain unclear. We hypothesized that Notch mechanosensitivity in VSMCs (Morrow et al., 2007; Loerakker et al., 2018) can regulate this hypertensive thickening. To test this hypothesis, we developed a computational framework coupling a FE analysis of arterial mechanics to an AB model of mechanosensitive Notch signaling. This one-way coupling enabled a more accurate analysis of Notch signaling in arteries compared to previous models (Loerakker et al., 2018; Ristori et al., 2020), accounting for the potential local heterogeneity of the arterial wall stretch when the homeostatic mechanical state is perturbed. Our simulations revealed that sensitivity of Notch to stretch at mean blood pressure can explain arterial thickening in response to hypertension, and that a loss of residual stress and Notch manipulations can strongly influence this adaptation.

Our prediction that the stretch at mean blood pressure is a likely target variable for Notch signaling in VSMCs can enhance our overall understanding of arterial G&R. It is generally accepted that arteries grow and remodel in response to mechanical stimuli to maintain some target variable(s) close to (a) preferred, homeostatic value(s) (Humphrey, 2008; Cyron and Humphrey, 2017; Humphrey and Schwartz, 2021). Even though stress and strain have been suggested as target variables (Humphrey, 2008; Eichinger et al., 2021), there is no consensus over the pressure at which these quantities should be calculated. For example, previous studies have related arterial G&R in hypertension to systolic pressure (Matsumoto and Hayashi, 1994; Latorre and Humphrey, 2020; Latorre et al., 2021; Spronck et al., 2021), mean pressure (Humphrey and Rajagopal, 2003; Baek et al., 2006; Karšaj et al., 2010; Cyron and Humphrey, 2014; Braeu et al., 2017, 2019; Latorre and Humphrey, 2018), and pulse pressure (Lacolley et al., 2009; Eberth et al., 2010). Inspired by this, here we compared three hypotheses, namely that Notch signaling in arteries is responsive to either systolic stretch, mean stretch, or dynamic stretch. Three types of hypertension were simulated, each of which is known to result in arterial wall thickening (Al-Nimer et al., 2010; Chen et al., 2011; Manios et al., 2015; Monzo et al., 2021). Given the key role of synthetic VSMCs in G&R (Owens et al., 2004; Rensen et al., 2007; Frismantiene et al., 2018), we assumed that arterial thickening would occur only when the simulations predicted a shift to synthetic VSMCs in the arterial wall. Such a phenotypic switch was predicted for all three types of hypertension only when Notch was assumed to depend on mean stretch (Figs. 3H, 4H and 5H). Therefore, our simulations suggest this quantity as the most likely target variable for Notch signaling. However, since the mean stretch was determined with respect to the stress-free configuration (eq. (6)) and the material properties were homogeneous, stretch and intramural stress were monotonically related in our simulations. Therefore, it was not possible to determine whether mean stretch or mean stress acts as the actual target variable. Nevertheless, this outcome brings us closer to determining the target variable(s) for arterial G&R and suggests that it may be stretch or stress at mean blood pressure, consistent with the assumption commonly made in computational G&R frameworks (Humphrey and Rajagopal, 2003; Baek et al., 2006; Karšaj et al., 2010; Cyron and Humphrey, 2014; Braeu et al., 2017, 2019). However, other variables, such as stress or strain at systolic or pulse pressure, should not be ruled out as potential targets for other biological phenomena mediating arterial G&R (Koffi, 1999; Boutouyrie et al., 1999; Eberth et al., 2009, 2010), in addition to Notch signaling. This is also reflected in the trend of more recent computational G&R studies that use systolic stress as target for arterial G&R (Latorre et al., 2019, 2021; Latorre and Humphrey, 2020; Spronck et al., 2021).

Arteries are well known to thicken in response to hypertension (Wolinsky, 1970; Vaishnav et al., 1990; Matsumoto and Hayashi, 1994; Hayashi and Sugimoto, 2007). Notch signaling regulates this process (Li et al., 2009; Qiao et al., 2012; Ragot et al., 2016), but the underlying mechanisms have remained unclear. Our simulations showed that Notch mechanosensitivity can explain arterial thickening in response to hypertension (Fig. 3B,E,H, 4B,E,H, and 5B,E,H). Supporting this conclusion, our model also predicted that G&R activity was highest in arteries subjected to CH (Fig. 5H), followed by ISH (Fig. 3H) and IDH (Fig. 4H), which agrees with experimental observations (Manios et al., 2015; Monzo et al., 2021). These results suggest the hypothesis that Notch mechanosensitivity is involved not only in the establishment of arterial homeostasis (Loerakker et al., 2018), but also in its maintenance.

In contrast to our computational results for coronary arteries (Figs. 3E, 4E and 5E), previous experiments in pulmonary arteries have shown an increase in Notch3 expression driving arterial thickening in hypertension (Li et al., 2009; Qiao et al., 2012). This suggests that the role and mechanosensitive behavior of Notch in hypertension may depend on the location in the vasculature, which would be in line with the highly context- and tissue-specific nature of Notch (Stassen et al., 2020; Karakaya and Van Asten et al. 2022). To our knowledge, Notch mechanosensitivity in VSMCs has only been investigated in the systemic circulation (Morrow et al., 2005; Loerakker et al., 2018). In coronary arteries, Notch has been shown to play a critical role in vascular adaptation to hypertension, as demonstrated by a lack of media hypertrophy in Notch3 knockout mice (Ragot et al., 2016). However, the changes in Notch activity were not monitored in this study. Further experimental studies on coronary arteries are therefore necessary, also towards validation of the computational results.

Our results suggest that manipulating Notch signaling may be an attractive therapeutic strategy in hypertension. Indeed, Notch is recognized as a potential target for the treatment of hypertension (Morris et al., 2019) and other cardiovascular diseases (Zhou et al., 2015; Zhu et al., 2017; Davis et al., 2018; Ristori et al., 2021). We specifically simulated the effects of over- or underexpression of Notch receptors and Jagged ligands. The model predicted that changes in Notch expression, but not Jagged expression, had a considerable influence on Notch activity. This may enable the control of VSMC phenotype to either intensify or counteract the effects of hypertension. Our results for a decrease in Notch expression (Fig. 8A) agree with previous studies showing a shift to synthetic VSMCs (Ragot et al., 2016) and a loss of contractile capabilities (Boulos et al., 2011) in Notch3 knockout mice. However, complete inhibition of Notch signaling by either Notch3 knockouts or γ-secretase inhibition was shown to result in less hypertrophy and wall thickening in hypertensive arteries (Li et al., 2009; Qiao et al., 2012; Ragot et al., 2016). A possible explanation for this is that Notch3 signaling was blocked completely in these studies, which might have led to cell apoptosis (Li et al., 2009; Qiao et al., 2012; Ragot et al., 2016) and arterial wall rarefaction (Ragot et al., 2016) rather than thickening. Our results could therefore be validated with transducible or conditional transgenic mouse models or *in vitro* with overexpression or knockdown via viral transduction (Driessen et al., 2018), rather than complete inhibition.

An increased control over tissue G&R via Notch manipulations may also aid in improving tissue engineered blood vessels and heart valves (Carlson et al., 2007; Zohorsky and Mequanint, 2021; Tiemeijer et al., 2022; Karakaya and Van Asten et al. 2022). Such manipulations may consist of immobilizing external Jagged ligands to scaffolds (Beckstead et al., 2006; Putti et al., 2019a, 2019b), a technique that has previously been found beneficial for tissue development (Beckstead et al., 2006; Wen et al., 2014; Dishowitz et al., 2014). In future studies, the present model could be extended to include the effects of external ligands, enabling the systematic optimization of scaffold design and tissue engineering protocols to reduce the need for experimental trial-and-error approaches.

The new framework also enables the prediction of the effects of various isolated mechanical perturbations on VSMC phenotype and arterial G&R. For example, a loss of axial residual stress was predicted to result in a general shift of VSMCs to a more synthetic phenotype (Fig. 6G), while a loss of circumferential residual stress led to a more heterogeneous phenotype distribution over the arterial wall, with an increase in G&R activity on the luminal side (Fig. 6H). Such a loss of residual stresses can occur during aging, as residual stresses mainly depend on the presence of elastin (Davis, 1995; Cardamone et al., 2009) and the elastin content in aged arteries is reduced (Greenwald, 2007; O’Rourke, 2007). Aging is generally accompanied by thickening of the arterial wall (O’Rourke, 2007; Thijssen et al., 2016) which fits with our prediction of a more synthetic VSMC phenotype upon losing residual stresses. Furthermore, when a loss of axial residual stress was combined with hypertension, our simulations revealed a synergistic effect (Fig. 3). A similar outcome was observed when hypertension was superimposed on aging in mice and caused additional arterial thickening (Spronck et al., 2020). However, the process of aging is multifaceted and it is often difficult to distinguish the effects of aging from other factors (O’Rourke, 2007). Additionally, our model only captures one aspect of arterial aging and does not, for example, include the effects of arterial stiffening (O’Rourke, 2007; Thijssen et al., 2016; Lacolley et al., 2020) which has a significant impact on arterial mechanics as well. Therefore, it is unclear whether the experimentally observed effects are simply a result of a loss of residual stress. Nevertheless, our results suggest that a loss of residual stress might contribute to the effects of arterial aging and may be synergistic with hypertension.

The new model provides a more detailed analysis of arterial mechanics compared to previous models of Notch in arteries (Loerakker et al., 2018; van Engeland et al., 2019; Ristori et al., 2020), where a homogeneous strain distribution and a linear stress-strain relationship were assumed. In particular, the new model includes a FE analysis with a hyperelastic, fiber-reinforced material model (Oomen et al., 2018), with parameters fitted to experimental data of porcine coronary arteries (Van Den Broek et al., 2011), and residual stresses in both axial and circumferential directions. This results in a more detailed characterization of the arterial mechanical state, which can account for a potentially heterogeneous stretch distribution over the arterial wall such as the one seen in hypertension (Fig. 2). It thus enables a more accurate analysis of local mechanosensitive Notch signaling and VSMC phenotypes not only during hypertension, but also in other diseases and regeneration events. A sensitivity analysis (Fig. S2) revealed only marginal changes in the predicted NICD content and thus VSMC phenotype upon variations in the input parameters of the FE model, demonstrating the robustness of the model predictions. The level of detail could be further increased in future models by fitting the model parameters to layer-specific data of human arteries (Holzapfel et al., 2005). Additionally, a full feedback loop between Notch signaling, tissue G&R, and mechanics could be included in future models to capture the complete process of mechano-regulated, Notch-mediated vascular G&R, similar to existing multiscale models of other signaling pathways (Aparício et al., 2016; Keshavarzian et al., 2018; Irons et al., 2021). Such a two-way coupling seems to be feasible considering the limited computational time needed for both the FE and AB simulations. Finally, although individual parameter values were derived from experimental studies, it would be good to validate the computational Notch model which is currently very challenging as quantitative experimental data on mechano-regulated Notch signaling in VSMCs are largely missing. Overall, the framework overcomes one of the main limitations of previous studies and opens up new possibilities for the investigation of mechanosensitive Notch signaling in the cardiovascular system (Stassen et al., 2020).

In conclusion, a computational framework was developed that couples detailed arterial mechanics to mechanosensitive Notch signaling in coronary arteries to predict the phenotype of VSMCs in the arterial wall. Our results suggest that Notch mechanosensitivity to strain at mean blood pressure can be a key mediator of arterial G&R in response to hypertension. Simulations further showed that changes in the expression of Notch receptors may be employed to modify VSMC phenotype and thereby control arterial G&R. These findings can improve our understanding of the cellular mechanisms driving arterial G&R and adaptation in hypertension and identify the Notch pathway as a promising target for intervention in treatment and tissue engineering. Finally, this study provides a stepping stone for developing more mechanistic computational models of arterial G&R, which could in the future incorporate a complete feedback loop between Notch signaling and G&R.

## Supplementary Material

Appendix A. Supplementary data

## Figures and Tables

**Table 1:** Parameter values of the computational framework. The third and sixth columns report whether the model parameters were fitted in the present study or justified according to literature and previous experimental studies.

Arterial mechanics				Notch signaling		
Symbol	Value	Origin		Symbol	Value	Origin
*r*_*i*,0_	1.5 mm	Estimated from (Van Den Broek et al., 2011), see section 2.1.4		[*N_r_*, *J_pr_*]	[1400, 1600]	(Boareto et al., 2015; Loerakker et al., 2018) based on (Amsen et al., 2004)
*T*_0_	0.32 mm	2.1.4 (Van Den Broek et al., 2011)		*D_pr_*	100	(Loerakker et al., 2018) based on (High et al., 2007; Baeten and Lilly, 2017)
*φ_f_*	0.41	(Van Den Broek et al., 2011)		*γ*	0.1	(Boareto et al., 2015; Loerakker et al., 2018)
*N*	60	Oomen et al. (2018)		*γ_i_*	0.5	(Boareto et al., 2015; Loerakker et al., 2018) based on (Wang, 2011; Manderfield et al., 2012)
*μ*	95.4 kPa	Fitted (section 2.1.3)		[*Λ_N_*, *Λ_J_*, *Λ_D_*]	[2.0, 2.0, 0.0]	(Boareto et al., 2015; Loerakker et al., 2018) based on (Lewis, 1996; Sprinzak et al., 2010)
*k_1_*	2.31 kPa	Fitted (section 2.1.3)		[*n_N_*, *n_J_*, *n_D_]*	[2.0, 5.0, 2.0]	(Boareto et al., 2015; Loerakker et al., 2018) based on (Sprinzak et al., 2010)
*k*_2_	7.57	Fitted (section 2.1.3)		*I*_0_	200	(Boareto et al., 2015; Loerakker et al., 2018)
*α*	31.2°	Fitted (section 2.1.3)		*N_c_*	32	Computed (section 2.2.1)
*d*	0.059	Fitted (section 2.1.3)		*k_t_*	2.5 × 10 ^5^ h-^1^	(Boareto et al., 2015; Loerakker et al., 2018) based on (Sprinzak et al., 2010)
*v*	0.498	Oomen et al. (2018)		*k_c_*	5.0 × 10-^4^ h-^1^	(Boareto et al., 2015; Loerakker et al., 2018) based on (Sprinzak et al., 2010)
Φ	98.9°	Computed (section 2.1.3)		*A_N_*	– 5.79	Loerakker et al. (2018)
$\lambda_{a}^{z}$	1.39 ^1^	Computed (section 2.1.3)		*A_J_*	– 4.17	Loerakker et al. (2018)
$\lambda_{a}^{\theta }$	0.9715^−1^ (inside) 1.0247^−1^ (outside)	Computed (section 2.1.3)		*J_ec_*	4000	Loerakker et al. (2018)

## Data Availability

All data and computational codes are available at https://doi.org/10.4121/20237562.v1
