# Supplementary material for: Computational analysis of the role of mechanosensitive Notch signaling in arterial adaptation to hypertension
Source: J Mech Behav Biomed Mater. Author manuscript; Available in PMC 2022 Sep 29. (PMC7613661; doi:10.1016/j.jmbbm.2022.105325)
Supplement: Appendix A. Supplementary data [file EMS154572-supplement-Appendix_A__Supplementary_data.docx]

**Supplementary material**


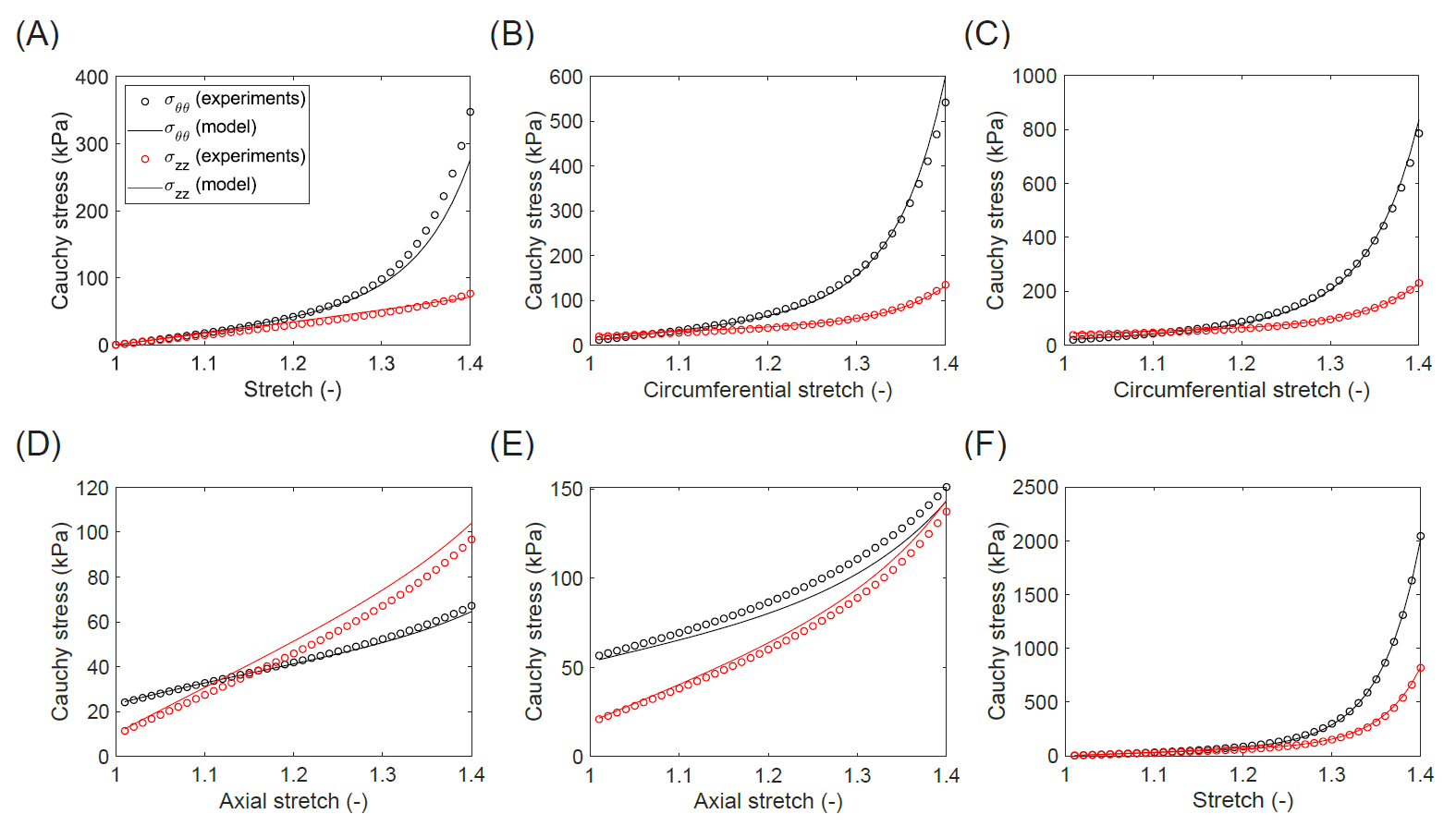


*Figure S1. Results of fitting the material parameters of the constitutive model to experimental data of porcine coronary artery from the literature for various simulated tensile tests. (A) Simulated uniaxial tensile tests in both circumferential and axial directions. (B) A simulated biaxial tensile test in which the axial stretch was fixed at 1.1 and the circumferential stretch was varied. (C) A simulated biaxial tensile test in which the axial stretch was fixed at 1.2 and the circumferential stretch was varied. (D) A simulated biaxial tensile test in which the circumferential stretch was fixed at 1.1 and the axial stretch was varied. (E) A simulated biaxial tensile test in which the circumferential stretch was fixed at 1.2 and the axial stretch was varied. (F) A simulated equibiaxial tensile test in which both axial and circumferential stretch were varied simultaneously. Circles represent data points derived from a previously published constitutive model based on experimental data* (Van Den Broek et al. 2011) *and solid lines are the fitted curved from the present constitutive model.* $\sigma_{\theta\theta}$ *and* $\sigma_{zz}$ *are the normal components of the Cauchy stress in circumferential direction (black) and axial direction (red), respectively.*


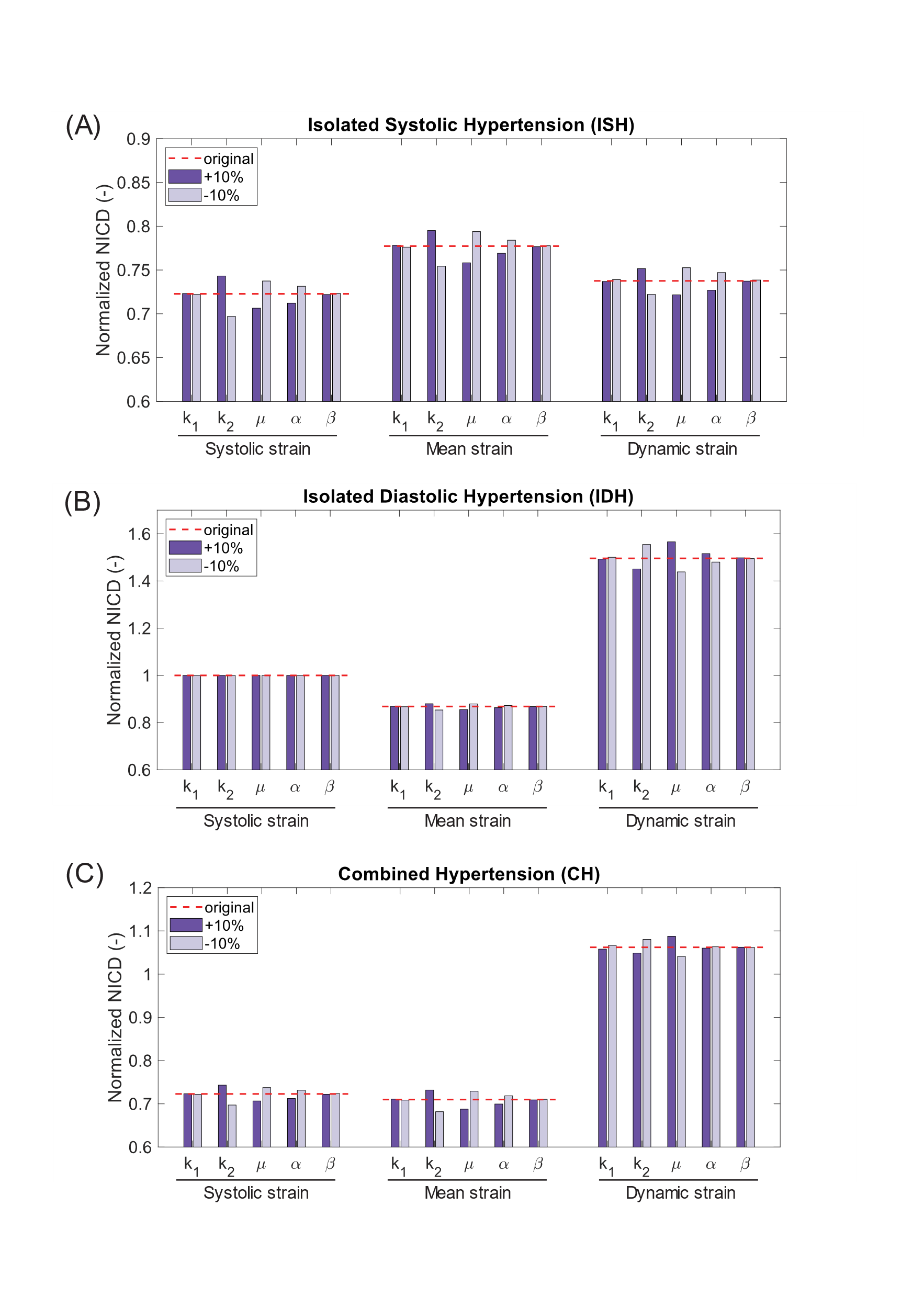


*Figure S2: Sensitivity Analysis of the coupled framework. Each of the fitted input parameters of the FE model was varied individually from -10% to 10% of its original value (as given in Table 1). The most severe case of each type of hypertension (A. ISH: 180/80 mmHg, B. IDH: 120/110 mmHg, and C. CH: 180/110 mmHg) was simulated and the corresponding NICD content was calculated. This NICD content was normalized to the NICD content of a normotensive artery (120/80 mmHg) which was simulated using the same parameter variation. The normalized NICD content was determined using the three hypotheses for the target variable of Notch: systolic strain, mean strain, and dynamic strain. The results are compared to those of the original simulations (Figures 3-5) represented by the dotted line.*
